# Can support from the father of the baby buffer the adverse effects of depressive symptoms on risk of preterm birth in Black families?

**DOI:** 10.3934/publichealth.2018.1.89

**Published:** 2018-03-30

**Authors:** Carmen Giurgescu, Lara Fahmy, Jaime Slaughter-Acey, Alexandra Nowak, Cleopatra Caldwell, Dawn P Misra

**Affiliations:** 1 College of Nursing, The Ohio State University, Columbus; 2 Department of Family Medicine & Public Health Sciences, School of Medicine, Wayne State University, Detroit; 3 College of Nursing & Health Professions, Drexel University, Philadelphia; 4 Department of Health Behavior and Health Education, School of Public Health, University of Michigan, Ann Arbor

**Keywords:** preterm birth, depressive symptoms, social support, father of the baby, pregnancy, Blacks

## Abstract

**Background:**

While maternal depressive symptoms during pregnancy have been linked to preterm birth (PTB; birth before 37 completed weeks of gestation), little has been reported on potential buffering factors, particularly specific to Black women who are at much higher risk. We examined the association between depressive symptoms and PTB in pregnant Black women, with father of the baby (FOB) support as a potential buffering factor.

**Methods:**

Data were obtained from the life-course influences on fetal environments study (2009–2011), a cohort of 1,410 Black women in metropolitan Detroit, Michigan (71% response rate) using maternal interviews and medical record abstraction collected during the postpartum hospitalization. The 20-item Center for Epidemiologic Studies Depression (CES-D) scale was used to measure depressive symptoms. The 14-item social networks in adult relations questionnaire was used to assess the mother's relationship with the FOB. Logistic regression was used to explore the interaction between CES-D and FOB support with regard to PTB risk. We adjusted for maternal advanced age, income, education level, smoking status, hypertension, prenatal care and BMI.

**Results:**

The PTB rate in this cohort was 17.7%. Among women with FOB scale < 60 (less support), the odd ratio (OR) of PTB for women with CES-D scores ≥ 23 (severe depressive symptoms) as compared to CES-D scores < 23 (no severe depressive symptoms) was 2.57 [95% confidence interval (CI): 1.68, 3.94; *p* < 0.001]. Among women with FOB scores ≥ 60 (more support), the odds of PTB in women with CES-D scores ≥ 23 did not significantly differ from the odds of PTB in women with CES-D scores < 23 (OR = 1.34; 95% CI: 0.74, 2.44; *p* = 0.3). After adjustment for covariates, among women with FOB scores < 60, the OR of PTB for women with CES-D scores ≥ 23 compared to < 23 was 2.79 (95% CI: 1.75, 4.45; *p* < 0.001). Among women with FOB scores ≥ 60, the odds of PTB in women with CES-D scores ≥ 23 was not statistically significantly different compared to the odds of PTB in women with CES-D scores < 23 (OR = 1.21; 95% CI: 0.62, 2.35; *p* = 0.6). The interaction term was statistically significant (*p* = 0.04).

**Discussion/Conclusions:**

The adverse effect of depressive symptoms on risk of PTB may be buffered by factors such as a supportive relationship with the FOB.

## 1. Introduction

Preterm birth (PTB; birth before 37 completed weeks of gestation) is the leading cause of infant mortality among Blacks [1]. Compared with non-Hispanic white women, Black women are more likely to have PTB (8.9% and 13.4%, respectively) [2]. Maternal depressive symptoms have received considerable attention with regard both to the etiology of PTB as well as an explanation for Black-white disparities in adverse birth outcomes [3]–[15]. Black women with higher levels of depressive symptoms are at higher risk of PTB [16]–[18]. Pregnant Black women also report lower levels of social support than pregnant white women [19]. Lack of social support was related to higher levels of depressive symptoms among Black women [20],[21]. A recent meta-analysis reported an increased risk for PTB (OR = 1.52; 95% CI: 1.18, 1.97) among women with high levels of stress and low levels of social support, suggesting a buffering effect of social support [22].

The maternal-paternal relationship, specifically support and conflict, may influence maternal depressive symptoms and affect birth outcomes. Black women whose father of the baby was supportive during pregnancy had lower levels of depressive symptoms compared with women whose father of the baby was not supportive during pregnancy [23]. In a sample of majority non-Hispanic white women (94%), depressed women who rated their partners as less supportive had babies who were born earlier compared with depressed women with higher perceived partner support [24]. Dissatisfaction with partner relationships has been related to psychological distress among pregnant women [25]. Among unmarried mothers, those in poor relationships were more likely to have depressive symptoms compared with women in good relationships [26]. The relationship with the father of the baby can be a source of support or conflict for the pregnant woman; therefore, the quality of the father-mother relationship has implications for birth outcomes. Thus, the purpose of this paper was to examine if father of the baby support moderated the associations between depressive symptoms and PTB among Black women.

## 2. Materials and methods

### 2.1. Design and sample

This present study is a secondary analysis of a retrospective cohort study of Black women participating in the life-course influences on fetal environments (LIFE) study conducted in the Detroit metropolitan area [20],[27]. Women were included in the LIFE study if they self-identified as Black or African American, were 18 to 45 years old and were within three days postpartum. Women were recruited from labor and delivery and postpartum units of a Detroit suburban hospital from June 2009 to December 2011. The main purpose of the original study was to examine the impact of racism on PTB for Black women [28]. A description of the methods for this study is provided in detail elsewhere [20],[27]. The study sample included 1,410 women, which represented 71% of the women approached for study participation.

### 2.2. Variables and instruments

Maternal characteristics. Maternal characteristics were measured based on participants' self-report (e.g., age, level of education, household annual income) and medical records (e.g., hypertensive disorders, month of pregnancy at the time of the first prenatal visit, number of prenatal care visits). The adequacy of prenatal care utilization was measured using the Kotelchuck Index based on (a) the adequacy of initiation of prenatal care measured as pregnancy months 1 and 2, months 3 and 4, months 5 and 6, and months 7 to 9; (b) the ratio of recorded prenatal visits compared to American College of Obstetrics and Gynecology recommended visits. Adequate prenatal care was a score of 80% or greater on the Kotelchuck Index. Pre-pregnancy Body Mass Index (BMI) was calculated based on maternal reported height and weight prior to pregnancy. We used the classification for BMI as underweight (< 18.5 kg/m^2^), normal weight (18.5–24.9 kg/m^2^), overweight (25–29.9 kg/m^2^) and obese (≥ 30 kg/m^2^) [29].

Depressive symptoms. The Center for Epidemiologic Studies Depression Scale (CES-D) [30] measures the presence of salient symptoms of depression within the past seven days (e.g., bothered by things more than usual, felt lonely). The CES-D has 20 items each rated on a 4-point scale referring to frequency of symptoms (0 = rarely, 1 = some of the time, 2 = occasionally, 3 = most of the time) with a total possible score ranging from 0–60. CES-D scores ≥ 23 have been correlated with major depression diagnosis [31],[32]. In the current secondary data analysis, the CES-D was used as a dichotomous variable (CES-D scores < 23, CES-D scores ≥ 23). Internal consistency reliability of the 20-item CES-D measure was 0.85.

Father of the baby support. The father of the baby (FOB) support was measured using the social networks in adult relations questionnaire [33],[34]. The tool has 14 items [e.g., (FOB) is always there when I need him; I feel that I can tell (FOB) just about everything; (FOB) is someone I can count on for financial support if I need it; (FOB) is someone I can count on to take care of my baby] on a 5-point scale (strongly disagree to strongly agree). The total possible score ranges from 14–70. There is no standard handling of the score of this construct. We used the median as a simple approach for contrast. We split the instrument at the median score for our sample and used a dichotomous variable of FOB support: Supportive (scores 60 or higher) and not supportive (scores less than 60). We were also interested a priori in considering context (interactions) and knew that statistical power might be too low if we proceeded to use quartiles or quintiles as well as be more difficult to interpret.

Preterm birth. Gestational age at birth was collected from medical records. Gestational age was computed based on the reported start date of the participant's last menstrual period (LMP) and corroborated using ultrasound measurements of the crown-rump length [35],[36]. In the case there was inconsistency between LMP and the ultrasound, the estimate based on the early ultrasound was used (n = 460). If both the ultrasound and LMP were missing, we used the provider's estimate from a late ultrasound (after 20 weeks gestation) (n = 165), the provider's estimate of gestation at birth (n = 61 women), or gestational age from the birth records, if everything else was missing (n = 21). PTB was measured as gestational age at birth of less than 37 completed weeks of gestation.

### 2.3. Data analysis

Descriptive statistics were used to describe maternal characteristics, CES-D and FOB scale. Chi-square was used to examine differences in sample characteristics between women with PTBs and women with term births. Binary logistic regression was used to examine if FOB support moderated the associations between depressive symptoms and PTB. We examined a number of potential confounders hypothesized to relate to depressive symptoms, FOB support and preterm birth: Advanced maternal age (<35 years of age *vs.* ≥35 years of age), low household income (< $20,000 *vs.* ≥ $20,000), low levels of education [< high school vs. graduated high school or completed high school equivalency test (e.g., GED)], smoking in the past year (yes *vs.* no), hypertensive disorders (e.g., chronic hypertension, preeclampsia) (yes vs. no), inadequate prenatal care (yes *vs.* no), and BMI (<18.5 kg/m^2^, 18.5–24.9 kg/m^2^, 25–29.9 kg/m^2^, ≥30 kg/m^2^).

## 3. Results

Majority of women were less than 35 years of age (85%), graduated high school or completed a high school equivalency test (92%), and had a household income of $20,000 or more (72%). Eight percent of women had hypertensive disorders. A subsample of 250 women had PTB (17.7%). Women had a CES-D mean of 15.31 ± 9.77 and a FOB scale mean of 55.47 ± 12.54. Twenty percent of women had CES-D scores ≥ 23 (data not shown). Women with PTBs were more likely to have hypertensive disorders and CES-D scores ≥ 23 compared with women with term births (see Table 1).

### 3.1. Unadjusted models

In an unadjusted logistic regression model, women who had CES-D scores ≥ 23 were 1.8 times more likely to have PTB compared with those with CES-D scores < 23 (OR = 1.82; 95% CI: 1.33, 2.50; *p* < 0.001). The risk of PTB did not vary by the mother's FOB scores ≥ 60 (above the median reflecting higher support) compared to those < 60 (OR = 1.03; 95% CI: 0.77, 1.36; *p* = 0.8). Based on prior work of our group and others, we explored whether there might be an interaction between depressive symptoms and FOB support with regard to PTB risk. Among women with FOB scale < 60, the OR of PTB for women with CES-D scores ≥ 23 compared to < 23 was 2.57 (95% CI: 1.68, 3.94; *p* < 0.001). In other words, among women with FOB scores < 60, women with CES-D scores ≥ 23 had a 157% (95% CI: 68, 294; *p* < 0.001) increased odds of PTB compared to women with CES-D < 23. Among women with FOB scores ≥ 60, the odds of PTB in women with CES-D scores ≥ 23 was not statistically significantly different compared to the odds of PTB in women with CES-D scores < 23 (OR = 1.34; 95% CI: 0.74, 2.44; *p* = 0.3).

**Table 1.** Sample characteristics^a^.

Variable	Full term birth N (%)	Preterm birth N (%)	χ^2^
Maternal Age			1.866
< 35 years	990 (82.8)	205 (17.2)	
≥ 35 years	169 (79.0)	45 (21.0)	
Level of Education			0.241
< 12 years	1059 (82.1)	231 (17.9)	
≥ 12 years	99 (83.9)	19 (16.1)	
Household Annual Income			0.180
< $20,000	289 (81.2)	67 (18.8)	
≥ $20,000	739 (82.2)	160 (17.8)	
Smoking in the Past Year			0.172
Yes	190 (81.2)	44 (18.8)	
No	960 (82.3)	206 (17.7)	
Inadequate Prenatal Care			0.323
Yes	589 (81.7)	132 (18.3)	
No	570 (82.8)	118 (17.2)	
Hypertensive Disorders			19.901**
Yes	70 (66.0)	36 (34.0)	
No	1038 (83.4)	207 (16.6)	
Body Mass Index			2.312
< 18.5	27 (79.4)	7 (20.6)	
18.5–24.9	368 (81.4)	84 (18.6)	
25–29.9	319 (84.8) 415 (81.5)	57 (15.2)	
≥ 30		94 (18.5)	
CES-D scores^b^			13.962**
< 23	909 (84.3)	169 (15.7)	
≥ 23	207 (74.7)	70 (25.3)	
FOB scores^c^			0.029
< 60	526 (81.9)	116 (18.1)	
≥ 60	522 (81.6)	118 (18.4)	

**p < 0.01.

^a^: The sample sizes are different for individual variables due to missing data.

^b^: CES-D: Center for Epidemiologic Studies Depression Scale.

^c^: FOB: Relationship with the father of the baby as measured by the Social Networks in Adult Relations Questionnaire.

### 3.2. Adjusted model

We found that advanced maternal age, low levels of education, low household annual income, smoking in the past year, hypertensive disorders, inadequate prenatal care, and BMI were confounders of the associations (change in regression coefficients of 10 percent or more). In a model adjusting for those confounders, among women with FOB scores < 60, the OR of PTB for women with CES-D scores ≥ 23 compared to < 23 was 2.79 (95% CI: 1.75, 4.45; *p* < 0.001). In other words, among women with FOB scores < 60, women with CES-D scores ≥ 23 had nearly 3 times the odds (2.79) of PTB compared to women with CES-D scores < 23. Among women with FOB scores ≥ 60, the odds of PTB in women with CES-D scores ≥ 23 was not statistically significantly different compared to the odds of PTB in women with CES-D scores < 23 (OR = 1.21; 95% CI: 0.62, 2.35; *p* = 0.6). The interaction term was statistically significant (*p* = 0.04) suggesting that the association between depressive symptoms and PTB in women with FOB scores < 60 was not the same as it was in women with FOB scores ≥ 60.

## 4. Discussion

We found that among women whose fathers of their babies were less supportive (FOB scores < 60), women who had CES-D scores ≥ 23 were more likely to have PTB compared with women who had CES-D scores < 23. Among women whose fathers of their babies were more supportive (FOB scores ≥ 60), the risk of PTB for women who had CES-D scores ≥ 23 was not different than the risk of PTB for women who had CES-D scores < 23. These results suggest that maternal depressive symptoms play a major role in increasing the risk for PTB among women with less support from the fathers of their babies. However, among women who have more support from the fathers of their babies, maternal depressive symptoms do not increase the risk for PTB.

Studies of birth outcomes typically have focused on exposures and experiences of the pregnant woman in isolation without considering her partner or family. We posit that research on birth outcomes needs to go beyond the focus of the pregnant woman and consider the role of the father. Why should Black fathers matter? [37] A recent meta-analysis reported that paternal Black race is a risk factor for adverse birth outcomes above and beyond maternal Black race; and Black mother-Black father couples were two times more likely to have PTB compared with white mother-white father couples [38]. In 2014, 71% of births to Black women occurred outside of marriage, compared with 29% of births to white women. Black fathers are an understudied group and are stereotyped as uninvolved with their children and not concerned about their parental role. However, data shows that Black fathers are involved with their children even when the relationship with the mother does not last [39]. The few studies that have explored paternal effects on birth outcomes have generally excluded understanding the dynamic, complex, and often correlated maternal-paternal relationship or they have been limited to an examination of paternal age, occupation, or socioeconomic status given the significance placed on fathers' role as a provider [40]. These approaches ignore a wide range of factors that may influence birth outcomes. Depressive symptoms are risk factors for PTB among Black women [16]–[18]. Research suggests that among pregnant depressed women, perceived lack of support from partner increased the risk of earlier birth [24]. In contrast, support from partner during the pregnancy has been shown to be protective for low birthweight infants [41] including Black women [42]. Thus, research needs to focus on the role of the father of the baby on birth outcomes among Black women.

## 5. Limitations

This study has some limitations. This is a retrospective cohort study where women were enrolled after birth. Research suggests that depressive symptoms do not change across pregnancy [21],[43]; therefore, it is likely that women who had CES-D scores ≥ 23 in the week prior to birth also had higher CES-D scores early in pregnancy. It is also possible that women may have differentially reported their depressive symptoms based on the outcome of pregnancy, but our own work comparing prospective to retrospective cohorts as well as a number of studies on recall bias in case-control research on congenital malformations suggests that this is likely minimal [44]. The LIFE study did not include diagnostic mental health measures, which are considered the gold standard for mental health assessment. Since the CES-D is a screening tool, the instrument does not measure major depression diagnosis. However, CES-D scores ≥ 23 have been correlated with major depression [31],[32]. Despite these limitations, these findings suggest that FOB scores moderated the association between CES-D scores ≥ 23 and PTB.

## 6. Conclusions

Complex processes are at work in the lives of pregnant Black parents. Understanding mechanisms through which these processes unfold is imperative for articulating risk and protective factors influencing birth outcomes. Although the literature has identified a number of risk factors for adverse birth outcomes associated with mothers, little attention has been given to understanding the role of fathers on birth outcomes. We found that among women whose fathers of their babies were less supportive, women who reported severe symptoms of depression were more likely to have preterm birth. The buffering effect of father of the baby support on the association between depressive symptoms and preterm birth may be one mechanism by which the father of the baby influences birth outcomes among Black women. Understanding the role of the father of the baby on birth outcomes will provide the foundation for designing interventions to eliminate disparities in PTB among Black women.
